# Glutamate Concentration in the Superior Temporal Sulcus Relates to Neuroticism in Schizophrenia

**DOI:** 10.3389/fpsyg.2018.00578

**Published:** 2018-05-07

**Authors:** Johanna Balz, Yadira Roa Romero, Julian Keil, Florian Schubert, Bernd Ittermann, Ralf Mekle, Christiane Montag, Jürgen Gallinat, Daniel Senkowski

**Affiliations:** ^1^Department of Psychiatry and Psychotherapy, St. Hedwig Hospital, Charité – Universitätsmedizin Berlin, Berlin, Germany; ^2^Department of Biological Psychology, Christian-Albrechts-University Kiel, Kiel, Germany; ^3^Physikalisch-Technische Bundesanstalt, Berlin, Germany; ^4^Center for Stroke Research Berlin, Charité – Universitätsmedizin Berlin, Berlin, Germany; ^5^Department of Psychiatry and Psychotherapy, University Medical Center Hamburg-Eppendorf, Hamburg, Germany

**Keywords:** schizophrenia, glutamate, neuroticism, magnetic resonance spectroscopy, superior temporal sulcus

## Abstract

Clinical studies suggest aberrant neurotransmitter concentrations in the brains of patients with schizophrenia (SCZ). Numerous studies have indicated deviant glutamate concentrations in SCZ, although the findings are inconsistent. Moreover, alterations in glutamate concentrations could be linked to personality traits in SCZ. Here, we examined the relationships between personality dimensions and glutamate concentrations in a voxel encompassing the occipital cortex (OCC) and another voxel encompassing the left superior temporal sulcus (STS). We used proton magnetic resonance spectroscopy to examine glutamate concentrations in the OCC and the STS in 19 SCZ and 21 non-psychiatric healthy control (HC) participants. Personality dimensions neuroticism, extraversion, openness, agreeableness and conscientiousness were assessed using the NEO-FFI questionnaire. SCZ compared to HC showed higher glutamate concentrations in the STS, reduced extraversion scores, and enhanced neuroticism scores. No group differences were observed for the other personality traits and for glutamate concentrations in the OCC. For the SCZ group, glutamate concentrations in STS were negatively correlated with the neuroticism scores [*r* = -0.537, *p* = 0.018] but this was not found in HC [*r*(19) = 0.011, *p* = 0.962]. No other significant correlations were found. Our study showed an inverse relationship between glutamate concentrations in the STS and neuroticism scores in SCZ. Elevated glutamate in the STS might serve as a compensatory mechanism that enables patients with enhanced concentrations to control and prevent the expression of neuroticism.

## Introduction

A large proportion of people diagnosed with schizophrenia (SCZ) show a chronic progression of positive and negative symptoms, cognitive impairments, as well as social dysfunction (Millan et al., 2016). Aberrant neurotransmission involving the neurotransmitter glutamate likely contributes to the development and progression of this disorder (Javitt and Zukin, 1991; Coyle, 1996). The *glutamate hypothesis* posits changes in glutamatergic synapses and alterations in glutamatergic pathways in SCZ (Coyle, 2006; Javitt, 2010; Kantrowitz and Javitt, 2010; Poels et al., 2014a,b). In support of this hypothesis, various studies have suggested a link between glutamate and psychosis (Egerton et al., 2012; Wijtenburg et al., 2015; Treen et al., 2016).

Glutamatergic synapses are found in the entire brain. However, alterations in glutamate-mediated neurotransmission and glutamate concentrations in SCZ seem to be confined to specific brain regions (Gallinat et al., 2016). To date, only few studies with SCZ patients have examined glutamatergic neurotransmission in the temporal lobe (Atagün et al., 2015; Hugdahl et al., 2015). For example, Hugdahl et al. (2015) reported decreased glutamate levels in the temporal lobe in SCZ compared to HC. However, the authors also found increased glutamate concentrations in a subgroup of SCZ patients with auditory hallucinations. In addition, another study has measured brain metabolites in the left superior temporal cortex in small participant samples but did not find differences in glutamate concentrations between SCZ and HC (Atagün et al., 2015). The superior temporal cortex is crucially involved in the processing and integration of information from multiple sensory modalities (Maier et al., 2008; Keil and Senkowski, 2018). Moreover, processing in the STS has an impact on perception (Balz et al., 2016). Hence, it is possible that interindividual differences at the synaptic and systemic levels in the superior temporal cortex also shape personality traits (Lu et al., 2014; Ikeda et al., 2015; Tseng et al., 2015; Li et al., 2017; Riccelli et al., 2017). For example, one study in healthy adults revealed a positive correlation between neuroticism scores and cortical thickness in the STS (Riccelli et al., 2017). In addition, the study showed negative correlations between both neuroticism and extraversion scores with the STS surface area and cortical volume. Another study using EEG reported an increase in functional connectivity between the insular cortex and STS during the processing of unpleasant emotions (Ikeda et al., 2015). Moreover, the study revealed that neural responses to unpleasant emotional stimuli in the inferior temporal gyrus were higher in participants with high neuroticism compared to participants with low neuroticism scores. Thus, differences in the STS architecture as well as in information processing in this area seem to be related to personality traits.

Studies investigating the Big Five personality traits in SCZ have consistently shown enhanced neuroticism and diminished extraversion scores (Berenbaum and Fujita, 1994; Gurrera et al., 2000; Camisa et al., 2005; Boyette et al., 2013; Ohi et al., 2016). Personality traits in SCZ relate to clinical symptoms, cognitive deficits, and impaired social function (Herrán et al., 2006; Kirihara et al., 2012; Gurrera et al., 2014; Compton et al., 2015). In addition, personality traits affect both the onset and the progression of SCZ (Van Os and Jones, 2001; Gleeson et al., 2005; Lönnqvist et al., 2009). Furthermore, post-mortem studies have indicated that glutamatergic neurobiological mechanisms might contribute to the susceptibility to neuroticism (Hettema et al., 2006; Ohi et al., 2017). Hence, it may be that alterations in the glutamatergic system relate to the SCZ psychopathology, as expressed in altered personality traits.

Thus far, no study has examined the link between glutamate concentration and personality traits in the superior temporal lobe. Recent magnetic resonance spectroscopy (MRS) studies have suggested both increased and decreased glutamate concentrations in the occipital lobe of SCZ patients (Chang et al., 2007; Thakkar et al., 2017). However, others studies did not find such alterations (Goto et al., 2012; Marsman et al., 2014). While higher and lower glutamate concentrations have been frequently reported in various brain areas of patients compared to control participants (Marsman et al., 2013; Poels et al., 2014a; Wijtenburg et al., 2015; Merritt et al., 2016), the functional significance of these alterations for the SCZ pathology are not well understood. In this study we examined glutamate concentrations in the OCC and the left STS of SCZ and healthy control (HC) participants. We used proton MRS to examine glutamate concentrations. Additionally, we assessed the Big Five personality dimensions using the NEO-FFI questionnaire (Costa and McCrae, 1992). We computed correlations between glutamate concentrations and those personality dimensions that differed between groups. This was carried out separately for each voxel and group. Since findings in the literature are relatively inconsistent, we hesitated deriving specific hypotheses regarding alterations in glutamate concentration in SCZ for the two obtained voxels. For the personality traits, we expected higher neuroticism scores and lower extraversion scores in SCZ than in HC. Finally, we explored whether alterations in glutamate concentrations serve as a marker for personality traits, especially neuroticism and extraversion, in SCZ and HC.

## Materials and Methods

### Participants

Twenty-three patients with the diagnosis schizophrenia were recruited from outpatient units of the Charité – Universitätsmedizin Berlin. The ICD-10 criteria, as used in Germany to classify psychiatric disorders, were applied. In addition, 23 healthy control participants matched for age, education, gender, and handedness, were recruited for the study. As it was not possible to recruit an equal amount of male and female SCZ patients that fitted our inclusion criteria, we made sure to maintain the same ratio for the HC group. The study was conducted in accordance with the Declaration of Helsinki and approved by the ethics committee of the Charité – Universitätsmedizin Berlin. All participants were additionally screened for mental disorders with the German version of the Structural Clinical Interview for DSM-IV-TR (SCID; First et al., 2002). This was done to assure that there were no co-morbidities in the patient group and no psychiatric disorders in the control group. Three SCZ elected not to complete the MRS scan. Furthermore, MRS data could not be obtained from one SCZ and two HC due to scanner malfunction. These participants were excluded from further analysis. The clinical diagnosis was assessed by a senior psychiatrist at the recruiting institution. All participants provided written informed consent, had normal hearing, normal or corrected to normal vision, and no other neurological disorders, alcohol or substance abuse. A random sample of 40% of all participants underwent a multi-drug screening test. Severity of symptoms in SCZ was assessed with the Positive and Negative Syndrome Scale (PANSS; Kay et al., 1987). To test cognitive performance, the Brief Assessment of Cognition in Schizophrenia (BACS; Keefe et al., 2004) was administered. The five major personality dimensions (Neuroticism; Extraversion; Openness; Agreeableness; Conscientiousness) were assessed with the NEO-FFI questionnaire (Costa and McCrae, 1989, 1992). **Table 1** provides an overview on demographic data, cognitive performance, and clinical scores of the study participants.

**Table 1:** Demographic data, positive and negative syndromes, and cognitive scores in the study participants.

	Patients		Controls		Statistics	
	Mean	*SD*	Mean	*SD*	t-values	*p*-values
Age (years)	36.26	8.68	36.29	8.14	-0.008	0.993
Education (years)	11.00	1.63	11.24	1.61	-0.464	0.645
Illness duration (years)	8.74	5.39	–	–	–	–
Chlorpromazin Eq. (daily dosage/mg)	379.47	162.94	–	–	–	–
	
	***N***		***N***			
	
Gender (m/f)	12/7		14/7			
Handedness (r/l)	16/3		18/3			
Antipsychotic med.^∗^	19		–			
Amisulpride	6		–			
Aripiprazole	2		–			
Clozapine	5		–			
Olanzapine	5		–			
Quetiapine	2		–			
Risperidone	5		–			
Antidepressive med.	2		–			
Mirtazapine	1		–			
Paroxetine	1		–			
**BACS**						
Verbal Memory	40.16	11.75	49.38	10.12	-2.667	0.011
Digit Sequencing	18.63	4.07	20.95	3.98	-1.821	0.076
Token Motor	65.00	12.45	76.14	9.84	-3.156	0.003
Verbal Fluency	45.11	13.12	54.81	16.01	-2.084	0.044
Symbol coding	50.68	9.85	58.43	13.75	-2.028	0.050
Tower of London	17.32	3.15	17.81	2.36	-0.565	0.575
Total score	236.89	35.31	277.52	36.29	-3.581	0.001
**PANSS**						
Positive	10.37	2.27	–	–	–	–
Negative	15.53	2.52	–	–	–	–
Disorganization	7.74	1.82	–	–	–	–
Excited	8.37	.90	–	–	–	–
Depressed	8.11	1.10	–	–	–	–
**NEO – FFI**						
Neuroticism	25.63	6.34	15.90	7.49	4.407	0.000
Extraversion	21.74	4.68	27.62	3.85	-4.358	0.000
Openness	30.37	5.84	31.43	7.30	-0.504	0.617
Agreeableness	30.95	4.43	32.29	4.64	-0.931	0.358
Conscientiousness	32.26	6.13	34.67	5.70	-1.286	0.206

*The table depicts demographic data and BACS scores for SCZ and HC, as well as PANSS scores for SCZ. BACS, Brief Assessment of Cognition in Schizophrenia; PANSS, Positive and Negative Syndrome Scale. ^∗^Note that most patients received more than one antipsychotic medication, and two patients received additional antidepressive co-medication. No mood stabilizers, anticonvulsants or sedatives were prescribed.*

### MR Methods

**FIGURE 1:**
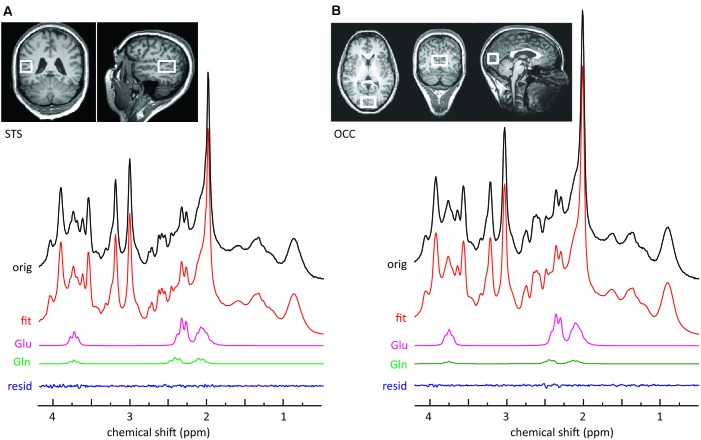
Sample MRS spectra from the left STS voxel **(A)** and the OCC voxel **(B)**. The upper panels illustrate the location of the voxel on T1-weighted images. The top row of each panel represents the original SPECIAL spectrum. Also shown are the overall fits and the fitted components of interest. The small residuals reflect the high quality of the fits.

MR images and spectra were collected on a 3 T Verio (Siemens Healthcare, Erlangen, Germany). Anatomical images were acquired using a three-dimensional T1-weighted magnetization prepared gradient-echo sequence (MPRAGE) with an isotropic resolution of 1.0 mm, a repetition time (TR) of 2.3 s, an echo time (TE) of 3.03 ms, an inversion time (TI) of 900 ms, and a flip angle of 9°. The volume of interest (VOI = 20 mm × 30 mm × 20 mm) for single voxel MRS, encompassing the left STS, was positioned below the upper bank of the temporal cortex (**Figure 1A**). The second VOI (30 mm × 20 mm × 20 mm) was positioned centrally, encompassing the occipital cortex (OCC) (**Figure 1B**). The transmitter radiofrequency (RF) voltage was calibrated for the individual VOI, followed by adjustment of first- and second-order shims using FAST(EST)MAP (Gruetter and Boesch, 1992; Gruetter and Tkac, 2000). Spin echo full intensity acquired localized (SPECIAL) (Mlynárik et al., 2006) spectra were acquired. The short echo time of the SPECIAL sequence enables the determination of a large number of metabolites and yields a high precision for the detection of glutamate (Mekle et al., 2009; Schubert et al., 2017). The sequence was used with water suppression by VAPOR (variable power radio frequency pulses with optimized relaxation delays; Tkac et al., 1999), and six outer volume suppression slices placed around the spectroscopic voxel to saturate outside spins. For each metabolite spectrum the number of acquisitions (NA) was 256 with TR = 3 s and TE = 8.5 ms. Following this scan, a spectrum without water suppression was recorded (NA = 8).

If the water line width calculated from a fit to the water spectrum after shimming was greater than or equal to 9.5 Hz, all corresponding MRS data were excluded from further analysis. The mean water line width (±standard deviation) of the remaining dataset (*n* = 40) was determined to be 8.5 (±1.3) Hz in STS and 7.6 (±1.1) in OCC. Linewidth did not significantly differ between SCZ and HC. For the analysis of the SPECIAL data, the spectra were corrected for frequency drift during acquisition and analyzed using LCModel (Provencher, 1993) with a simulated basis set containing 20 metabolites.

The unsuppressed water spectrum was used for eddy current correction and referencing the metabolite spectrum to the internal water concentration. Cramér-Rao lower bounds of the LCModel fits were 4.3 ± 0.7% for glutamate in the STS and 4.6 ± 1.3% for glutamate in the OCC (*n* = 40). Glutamate amplitudes were corrected for relaxation using T1 and T2 values determined at 3T (Schubert et al., 2004; Mullins et al., 2008). To correct the *in vivo* concentrations for the amount of cerebrospinal fluid (CSF) in the selected VOI, segmentation of the T1-weighted images was performed using statistical parametric mapping (SPM8; Litvak et al., 2011). Pixels in the VOI were classified according to their probability calculated by SPM8 to belong to one of the tissue types: CSF, gray matter, or white matter. The average glutamate concentrations calculated in this manner were 8.64 ± 1.27 mmol/l in STS and 7.68 ± 1.02 mmol/l in OCC.

### Data Analysis

For SCZ and HC, the NEO-FFI scores were evaluated for each personality dimension. In a first step, we compared glutamate concentrations in the STS and OCC between SCZ and HC. In this comparison we used a Bonferroni-corrected alpha-level of 0.025 (0.05/2). In the next step personality dimensions between both groups were compared using two-sample *t*-tests. Here, a Bonferroni-corrected alpha-level of 0.01 (0.05/5) was used. Significant group differences were followed up by analyses of Pearson correlation between glutamate concentrations and NEO-FFI scores. In this comparison we used a Bonferroni-corrected alpha-level of 0.025 (0.05/2) within SCZ and HC. Fisher *Z*-values were calculated to compare correlation coefficients between groups. In addition, if the analysis revealed significant correlations between glutamate concentrations and NEO-FFI scores, we investigated whether they were mediated by the factors age and gender in the control group and by the factors age, gender and medication in the patients group. To statistically control for these factors partial correlation analyses were conducted. In patients the medication dosage was converted to chlorpromazine equivalent (CPZE) level (Gardner et al., 2010). For exploratory purposes, in the SCZ group, glutamate values were also related to psychopathology scores (PANSS) using Pearson correlations.

## Results

**FIGURE 2:**
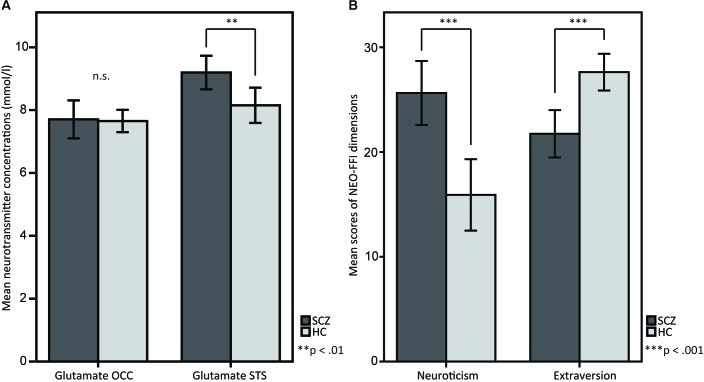
*T*-tests between SCZ and HC for **(A)** glutamate in the OCC and glutamate in the STS, and for **(B)** the NEO-FFI dimensions neuroticism and extraversion. **(A)** The barplots show OCC and STS mean glutamate concentrations in SCZ and HC. Glutamate concentrations in the STS are elevated in SCZ compared to HC, whereas OCC glutamate concentrations do not show any differences. **(B)** The barplots show differences between SCZ and HC in the personality dimensions neuroticism and extraversion, measured by the NEO-FFI.

SCZ showed higher glutamate concentrations in STS compared to HC [*M* = 9.193, *SD* = 1.106 vs. *M* = 8.151, *SD* = 1.231, *t*(38) = 2.806, *p* = 0.008; **Figure 2A**]. However, no significant differences were observed for occipital glutamate concentrations [*M* = 7.705, *SD* = 1.255 vs. *M* = 7.652, *SD* = 7.777, *t*(38) = 0.160, *p* = 0.874]. Across groups, there were no differences in glutamate concentrations between female and male participants [female (*n* = 14): *M* = 21.21, *SD* = 8.496 vs. male (*n* = 26): *M* = 20.15, *SD* = 8.582, *t*(38) = -0.374, *p* = 0.710]. The analysis of personality traits revealed enhanced neuroticism scores [*M* = 25.63, *SD* = 6.344 vs. *M* = 15.90, *SD* = 7.489, *t*(38) = 4.407, *p* < 0.001] and reduced extraversion scores [*M* = 21.74, *SD* = 4.677 vs. *M* = 27.62, *SD* = 3.853, *t*(38) = -4.358, *p* < 0.001] in SCZ compared to HC (**Figure 2B**). No significant group differences were found for the other personality traits [Openness: *M* = 30.37, *SD* = 5.842 vs. *M* = 31.43, *SD* = 7.298, *t*(38) = -0.504, *p* = 0.617; Agreeableness: *M* = 30.95, *SD* = 4.428 vs. *M* = 32.29, *SD* = 4.638, *t*(38) = -0.931, *p* = 0.358; Conscientiousness: *M* = 32.26, *SD* = 6.127 vs. *M* = 34.67, *SD* = 5.695, *t*(38) = -1.286, *p* = 0.206]. Across groups, no significant differences in neuroticism and extraversion scores were found between female and male participants [neuroticism: female (*n* = 14): *M* = 21.21, *SD* = 8.496 vs. male (*n* = 26): *M* = 20.15, *SD* = 8.582, *t*(38) = -0.374, *p* = 0.710; extraversion: female (*n* = 14): *M* = 25.29, *SD* = 5.239 vs. male (*n* = 26): *M* = 24.58, *SD* = 5.194, *t*(38) = -0.410, *p* = 0.684].

**FIGURE 3:**
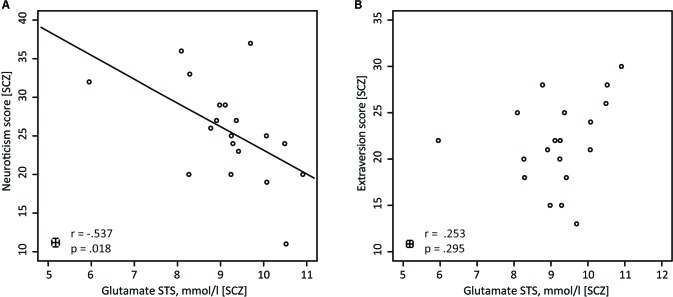
Pearson correlations for SCZ between glutamate (STS) and neuroticism, and between glutamate (STS) and extraversion. **(A)** In SCZ glutamate concentrations in the STS are negatively correlated with neuroticism (*n* = 19). **(B)** In SCZ glutamate concentrations in the STS do not show any correlations with the NEO-FFI extraversion score (*n* = 19).

In the next step of the analysis, personality traits that showed significant group differences, i.e., Extraversion and Neuroticism, were correlated with glutamate concentrations in the STS and OCC. This was done separately for the SCZ and HC groups. Within the SCZ group, the glutamate concentrations correlated negatively with the neuroticism scores [*r*(17) = -0.537, *p* = 0.018] (**Figure 3A**). Higher glutamate concentrations were related to lower neuroticism scores. However, no such correlation was found in HC [*r*(19) = 0.011, *p* = 0.962]. Importantly, the correlation coefficients for the relationships between neuroticism scores and glutamate concentrations in the STS differed significantly between groups [Fisher *Z* = -1.714, *p* = 0.043]. Next, we conducted a partial correlation analysis with the control variables age, gender and medication, i.e., CPZE level, between glutamate concentration in the STS and neuroticism scores in SCZ. The analysis revealed a significant relationship [*r*(14) = -0.518, *p* = 0.040], demonstrating that age, gender, and medication did not substantially mediate the negative correlation between glutamate concentration in the STS and neuroticism scores in SCZ.

Further analyses revealed that for both groups, there were no significant correlations between glutamate concentrations in the STS and extraversion scores [SCZ: *r*(17) = 0.253, *p* = 0.295 (**Figure 3B**); HC: *r*(19) = 0.394, *p* = 0.077]. In addition, the correlation analysis between glutamate concentrations in the occipital lobe and neuroticism scores [SCZ: *r*(17) = -0.412, *p* = 0.079; HC: *r*(19) = 0.136, *p* = 0.557], as well as between glutamate concentration in the occipital lobe and extraversion scores [SCZ: *r*(17) = -0.014, *p* = 0.954; HC: *r*(19) = -0.102, *p* = 0.659], did not reveal any significant relationships.

No relationship between glutamate and schizophrenia symptoms, as obtained through the PANSS, was found (for all scales *p* > 0.05). Moreover, there was no relationship between glutamate concentration and cognitive capacity, measured by the BACS (for all scales *p* > 0.05).

## Discussion

In this study we examined glutamate concentrations in the STS and OCC in SCZ and HC. We observed that glutamate concentrations in the STS were significantly higher for SCZ than HC, while there were no group differences in glutamate concentrations in the OCC. Additionally, we obtained measures of the Big Five personality traits. In line with previous research, we found increased neuroticism scores and reduced extraversion scores in SCZ compared to HC. Interestingly, within SCZ glutamate levels in the STS correlated negatively with neuroticism scores.

### Patients Exhibit Higher STS Glutamate Concentrations Than Healthy Controls

SCZ compared to HC showed higher glutamate concentrations in the STS, which is a key association area in the human brain. However, we did not find group differences in glutamate concentrations in the visual cortex. The lack of glutamate concentration differences between SCZ and HC in the visual cortex corresponds with a study by Marsman et al. (2014), who examined glutamate concentrations in the parieto-occipital cortex using a 7 T MRI scanner. The study revealed no significant group differences in glutamate concentrations in the parieto-occipital cortex. Previous studies measuring glutamate showed inconsistent results: Depending on the selected brain area, some studies did not find glutamatergic aberrations in SCZ (Marsman et al., 2013; Poels et al., 2014a,b; Wijtenburg et al., 2015; Merritt et al., 2016), while others did find both increased and decreased glutamate concentrations in SCZ (Marsman et al., 2013; Poels et al., 2014a,b; Wijtenburg et al., 2015; Merritt et al., 2016). In the STS, however, clear results have been missing so far. To our knowledge, only one previous study has examined glutamate concentrations in SCZ in the left STS in a small study sample (Atagün et al., 2015). The study did not reveal significant differences in glutamate concentrations between groups, perhaps due to a lack of statistical power. The results of the current study show, for the first time, increased glutamate concentrations in the left STS in SCZ compared to HC.

Recent research indicates that alterations in glutamatergic neurotransmission in SCZ do not start during the course of the disorder but are already prevalent before the visible manifestation of symptoms (Millan et al., 2016). For example, Egerton et al. (2012) suggested that genetic and environmental risk factors for psychosis can interact with glutamatergic neurotransmission and can consequently lead to the onset of SCZ. Furthermore, several studies have suggested that individuals at high-risk for developing a psychotic disorder show alterations in glutamate concentrations with lower glutamate concentrations in thalamic and higher glutamate concentration in medial frontal regions prior to the onset of SCZ (Tibbo et al., 2004; Stone et al., 2009; Fusar-Poli et al., 2011; Wijtenburg et al., 2015).

Generally, glutamate levels, e.g., as measured in thalamic regions and the anterior cingulate cortex, seem to be elevated in early stages of the psychosis as well as unmedicated first episode SCZ patients (Théberge et al., 2002; Egerton et al., 2012; Marsman et al., 2013; Wijtenburg et al., 2015). In contrast, the results in chronic, medicated patients are less clear (Egerton and Stone, 2012; Marsman et al., 2013; Poels et al., 2014a,b; Wijtenburg et al., 2015). Some studies have suggested that glutamate concentrations in various regions increase with age, or medication intake (Wijtenburg et al., 2015). However, in the present study we did not find an influence of age, or medication on glutamate concentrations in the left STS. The meta-analysis by Merritt et al. (2016) could also not confirm a relationship between glutamate concentration and age, antipsychotic treatment or symptom severity. Hence, our results suggest that glutamate concentrations do not seem to be directly related to those factors. Moreover, our study provides evidence that elevated glutamate concentrations in the STS are prevalent in SCZ even in medicated chronic patients.

### Patients Rate Themselves Higher in Neuroticism and Lower in Extraversion Than Healthy Controls

In line with our hypothesis, SCZ patients had higher neuroticism values and lower extraversion values than HC. Thus, our findings are in line with previous studies about personality trait differences in SCZ (Berenbaum and Fujita, 1994; Gurrera et al., 2000; Camisa et al., 2005; Boyette et al., 2013; Ohi et al., 2016). A number of studies have suggested that personality differences between SCZ and healthy individuals are present even before the onset of psychotic symptoms (Keshavan et al., 2005; Fryers and Brugha, 2013; Ohi et al., 2016). Furthermore, high neuroticism scores and low extraversion scores have been associated with an increased risk of developing SCZ (Van Os and Jones, 2001; Krabbendam et al., 2002; Goodwin et al., 2003; Gleeson et al., 2005; Krug et al., 2008; Lönnqvist et al., 2009; Boyette et al., 2013, 2015; Ohi et al., 2016). High neuroticism trait scores may reflect a vulnerability to distress, which could contribute to the development of SCZ (Nuechterlein et al., 1994; Boyette et al., 2013). Recently, Ridgewell et al. (2017) discovered a relationship between neuroticism and quality of life in SCZ. If neuroticism is constantly higher in individuals who are at high risk for developing SCZ, and if elevated neuroticism scores shape general health and the onset of SCZ, then elevated neuroticism trait scores could be a predictive marker for individuals who are at risk of developing SCZ or progressing to a psychotic phase. Future research should examine the predictive value of enhanced neuroticism scores for the development of SCZ.

Neuroticism and extraversion might be a manifestation of short-term symptoms that occur in SCZ. To test this assumption, Boyette et al. (2015) investigated the 3-year stability of the Big Five personality traits and found that transient psychotic symptoms and psychotic relapse did not substantially affect personality traits in patients with psychotic disorders. In addition, Kentros et al. (1997) failed to show a relationship between positive symptoms and personality trait scores in SCZ, although a slight influence of negative symptoms on personality traits was observed. Thus, these studies suggest that short-term symptoms in SCZ have a relatively modest impact on personality traits in SCZ.

When investigating personality traits in SCZ, one might argue that patients are unable to provide a reliable self-assessment on their personality. However, some studies have addressed this issue and found that SCZ patients have the same ability to rate their own personality as compared with healthy individuals (Bell et al., 2007). Bell et al. (2007) investigated the validity of self-report questionnaires for SCZ patients and found that standardized personality questionnaires, such as the NEO-FFI are valid even for patients with poor insight. Furthermore, Suslow et al. (2014) found that not only the explicit but also the implicit self-representation in SCZ is different from HC in the direction of higher neuroticism and lower extraversion in SCZ. Hence, we conclude that the personality traits measured in the current study provide a reliable measure of the Big Five personality dimensions in both study groups. Personality trait scores are relatively stable over time and do not seem to be related to the severity of the illness or to the current symptomatology of acute psychosis symptoms. Therefore, we argue that obtained alterations in personality trait scores, i.e., elevated neuroticism and diminished extraversion in SCZ reflect a trait rather than a state effect (Kentros et al., 1997; Gurrera et al., 2000; Beauchamp et al., 2006, 2011; Boyette et al., 2015).

### A Negative Correlation Between STS Glutamate Concentrations and Neuroticism Can Be Found in Schizophrenic Patients

In SCZ neuroticism scores, but not extraversion scores, correlated negatively with STS glutamate concentrations. The higher the STS glutamate concentration in SCZ, the lower the neuroticism scores were. Interestingly, glutamate concentrations in the STS were linked to neuroticism in SCZ, but not in HC. HC did not show significant correlations between STS glutamate and neuroticism or extraversion.

So far, only a few studies have discovered negative correlations between glutamate concentrations and symptoms of psychiatric disorders in SCZ. For example, Egerton et al. (2014), who examined individuals with ultra high risk of psychosis, found a negative relationship between thalamic glutamate concentration and the severity of positive symptoms – particularly the severity of abnormal thought content and the severity of perceptual abnormalities. Furthermore, longitudinal data in that study revealed a correlation between lower thalamic glutamate concentrations and successive worsening of symptoms over time. However, our study did not reveal any correlations of glutamate with PANSS or BACS ratings, which indicates that acute psychopathology is independent from glutamate concentration levels in the STS and the OCC. Notably, other studies also reported a lack of any significant relationship between acute psychotic symptoms and glutamate concentrations, e.g., in the anterior cingulate cortex or dorsolateral prefrontal cortex (Chiu et al., 2017; Ćurčić-Blake et al., 2017).

The observed effects in the current study were relatively weak and therefore require replication with a larger sample. Moreover, it is not clear if an enhancement in glutamate concentrations actually contributes to enhanced neural activity of glutamatergic neurons. Nevertheless, we could speculate that higher STS glutamate concentrations might be a compensatory mechanism that protects individuals with SCZ from otherwise higher neuroticism traits. Studies suggested that high neuroticism scores might have a negative impact on functioning and quality of life (Ridgewell et al., 2017). An elevated glutamate concentration in the STS in SCZ might therefore compensate the otherwise distinct personality differences and lead to better overall functioning. However, this assumption requires further testing.

Glutamate is the primary excitatory neurotransmitter in the human brain, hence, constantly elevated glutamate concentrations could lead to structural abnormalities and dangerous neurotoxic effects in the SCZ brain (Marsman et al., 2013; Plitman et al., 2016). Long-term studies are necessary to monitor changes in glutamatergic neurotransmission over time in SCZ and to check for damages caused by glutamatergic aberrations. If SCZ is linked to aberrant glutamatergic neurotransmission, then medication that can affect glutamatergic neurotransmission may be a promising psychopharmacological intervention target. The observation that glutamate is linked to and affects personality could be used as a treatment option to reduce symptoms of psychotic and other psychiatric disorders (Carlsson, 2001; Rosenberg et al., 2004; Hoerst et al., 2010; Neill and Frodl, 2012). However, since SCZ is a very heterogeneous disorder, it is possible that different types of treatment will be necessary to help to diminish the diverse symptoms in this patient group (Chavez-Noriega et al., 2002; Howes and Kapur, 2014; Poels et al., 2014b; Beck et al., 2016). Promising results supporting this therapeutic avenue propose that SCZ who do not respond to dopaminergic medication reveal normal dopaminergic levels, but altered glutamate levels (Demjaha et al., 2014). Consequently, drugs that target the glutamatergic system could help in individuals who do not respond to standard medication in SCZ, which mostly targets the dopaminergic system.

## Conclusion

Our study revealed that glutamate concentration in the STS and neuroticism in SCZ are linked. Patients with SCZ who exhibit higher glutamate concentrations in the STS show lower neuroticism and vice versa. We propose that glutamate could serve as a compensatory mechanism in the SCZ brain. Specifically, enhanced glutamate concentrations in the association cortex might enable patients to control and subsequently prevent the expression of neuroticism. Considering that modulations in neurotransmission also affect personality in SCZ, monitoring and moderating the glutamatergic concentration might help to better understand SCZ psychopathology. Consequently, addressing the glutamatergic dysfunction might help individuals with schizophrenia to overcome some of the limitations resulting from their higher neuroticism trait scores.

## Author Contributions

JB conceived the experiment, recorded data, analyzed data, discussed data, and wrote the manuscript. YRR conceived the experiment, recorded data, analyzed data, and wrote the manuscript. JK conceived the experiment, analyzed data, discussed data, and wrote the manuscript. FS and RM recorded data, analyzed data, and wrote the manuscript. BI analyzed data and wrote the manuscript. CM discussed the data and wrote the manuscript. JG conceived the experiment and wrote the manuscript. DS conceived the experiment, discussed data, and wrote the manuscript.

## Conflict of Interest Statement

The authors declare that the research was conducted in the absence of any commercial or financial relationships that could be construed as a potential conflict of interest.
